# Epidemiology, Burden, and Association of Substance Abuse Amongst Patients With Cardiovascular Disorders: National Cross-Sectional Survey Study

**DOI:** 10.7759/cureus.27016

**Published:** 2022-07-19

**Authors:** Nikhila Chelikam, Vandit Vyas, Lavanya Dondapati, Beshoy Iskander, Ghanshyam Patel, Siddhant Jain, Tanvi Singla, Ali Bombaywala, Daniel Zarrate, Neha Debnath, Nitesh K Jain, Appala Suman Peela, Urvish K Patel, Amit Sharma

**Affiliations:** 1 Clinical Research, Icahn School of Medicine at Mount Sinai, New York, USA; 2 Internal Medicine, Surat Municipal Institute of Medical Education and Research, Surat, IND; 3 Internal Medicine, Dr. Nandamuri Taraka Rama Rao (NTR) University of Health Sciences, Vijayawada, IND; 4 Internal Medicine, Bon Secours Mercy Health-St. Elizabeth Youngstown Hospital, Youngstown, USA; 5 Internal Medicine, Mercyhealth Internal Medicine Residency, Javon Bea Hospital, Rockford, USA; 6 Internal Medicine, Byramjee Jeejabhoy Medical College, Ahmedabad, IND; 7 Internal Medicine, Dayanand Medical College, Ludhiana, IND; 8 Department of Internal Medicine, Pravara Institute of Medical Sciences, Ahmednagar, IND; 9 General Medicine, El Bosque University, Bogota, COL; 10 Internal Medicine, Icahn School of Medicine at Mount Sinai, New York, USA; 11 Critical Care Medicine, Mayo Clinic Health System, Mankato, USA; 12 Family Medicine, University of North Carolina (UNC) Health Southeastern, Lumberton, USA; 13 Public Health and Neurology, Icahn School of Medicine at Mount Sinai, New York, USA; 14 Infectious Disease, Geisinger Community Medical Center, Scranton, USA

**Keywords:** cardiovascular disorders, nhanes, smoking, alcohol, substance abuse

## Abstract

Background: Substance use disorders (SUDs) are considered to be a major risk factor for cardiovascular disorders (CVDs). In 2019, as per the National Drug Use and Health Survey (NSDUH), 20.4 million American adults suffered from a substance use disorder. The main purpose of this study is to determine the prevalence of several SUDs (cigarette smoking, cigar, smokeless tobacco, marijuana, cocaine/heroin/methamphetamine, and injectable illegal drug) amongst patients diagnosed with various CVDs (angina pectoris, myocardial infarction, and coronary heart disease).

Methods: This is a retrospective cross-sectional study carried out using the National Health and Nutrition Examination Survey (NHANES) database from 2013 to 2018, and respondents with CVDs were recognized using questionnaires. Different SUDs (active history) were identified amongst the adult population with a history of CVDs and without CVDs. Univariate analysis was performed using chi-square and unpaired t-test/Mann-Whitney test to identify characteristics of respondents with CVDs and mix effect multivariable logistic regression models were generated to find the prevalence of SUDs amongst the CVD population. Datasets were analyzed using Statistical Analysis System (SAS) software, and the p-value of < 0.05 was considered statistically significant.

Results: Of the 263465 respondents, 7.90% respondents were diagnosed with CVDs and were noted to be in older age group (median age: 69 years). CVDs were more prevalent amongst 66-years and above (19.36% vs. 45-64 years: 6.81% vs. 18-44 years: 1.17%), male (10.40% vs. female: 5.66%), Non-Hispanic White race (10.92%), and lower annual household income population (<$25000 vs. >$100,000:12.21% vs. 4.01%) (p<0.0001). When compared with respondents without a history of CVDs, respondents with a history of CVDs were noted to be more prevalent with a concurrent diagnosis of hypertension (85.98% vs. 79.53%), hypercholesterolemia (68.78% vs. 34.54%), diabetes (37.86% vs. 12.70%), stroke (17.4% vs. 2.71%), and congestive heart failure (28.80% vs. 1.31%) (p<0.0001). History of CVDs were more prevalent amongst the respondents using marijuana (overall 53.14%; CVD vs. no-CVD 65.42% vs. 52.81%; p<0.0001), cigarette smoking (60.47% vs. 40.41%; p<0.0001), cigar-smoking (47.05% vs. 35.58%; p<0.0001), methamphetamine/cocaine/heroin (23.82% vs. 16.71%; p<0.0001), smokeless tobacco use (18.53% vs. 14.59%; p<0.0001), and injectable illegal drug use (4.67% vs. 2.43%; p<0.0001). Additionally, prevalence of history of CVDs was almost double in respondents using cigarettes without filters (2.28% vs. 1.10%; p<0.0001) when compared with respondents using cigarettes with filters.

Conclusion: Respondents who used marijuana or hashish, injectable illegal drugs, and e-cigars were at elevated risk for cardiovascular disorders. Providing situational awareness and offering a good support system can be a strategy to prevent the development of cardiovascular disorders among substance users.

## Introduction

The global burden of cardiovascular disorders (CVDs) has soared over the last few years. The year 2017 saw an increase in cardiovascular-related deaths by 21.1% as compared to that recorded in 2007 [1]. This increase is being attributed to lifestyle, food habits, and an increase in substance use disorders (SUDs), the most common being alcohol, tobacco smoking, e-cigarettes, stimulants, marijuana, steroids, and opioids. SUD is currently being regarded as major healthcare and social issue. According to the National Survey on Drug Use and Health (NSDUH), the prevalence of SUDs among individuals aged 12 or older has been escalating over the past decade, with 20.4 million cases reported in 2019 [2]. After relevant adjustment of other risk factors, studies suggest an increased incidence of acute cardiovascular emergencies like myocardial infarction with cocaine and/or marijuana use [3-5]. Overall mortality attributed to the consumption of tobacco in any form was as high as 8.7 million deaths in the year 2019, of which 36.7% were due to cardiovascular events [6]. Moderate alcohol consumption is considered to be cardioprotective; however cardiovascular mortality significantly increases in individuals who binge drink, i.e., >5 drinks per day in males and >4 drinks/day in females [7,8]. The impact of consumption of non-prescribed steroids on cardiovascular function is also being studied using modern-day technology. Many studies observed a significant change in left ventricular morphology along with a deranged lipid profile among individuals with frequent steroid use [9,10].

There is limited knowledge about the mechanism of how different substances impact cardiovascular health. However, few studies implicate multifactorial pathogenesis. Stimulants are associated with decreased catecholamine reuptake, thus causing sympathetic overdrive with increased myocardial oxygen demand and marked vasospasm along with abnormal platelet aggregation resulting in acute arterial hypertension, thrombosis, and accelerated atherosclerosis [11,12]. Similar to stimulants, marijuana also exerts its effect via increased sympathetic stimulation and depression of the parasympathetic system [13]. Analgesic properties of marijuana and opioids can lead to decreased awareness about cardiovascular symptoms, thus delaying the presentation of the patient to the hospital. Cigarette smoking has been acknowledged to generate oxidative stress on the arterial wall and also promote thrombus formation [14]. Researchers are also exploring the impact of advanced and effective nicotine delivery devices, popularly known as e-cigarettes, on cardiovascular health [15,16].

In the past decade, increased trends have been observed in the consumption of marijuana and methamphetamine, thus leading to increased mortality and morbidity among these substance users. Incidentally, a slight decrease in the use of cocaine was noted in the young patient population [2]. Complications of SUDs are preventable and reversible. Thus, American Heart Association (AHA) advises taking a detailed drug history in young patients presenting with cardiovascular events. This study is being conducted to see the point prevalence of various substance use in the population of age 12 years and older and with a positive history of cardiovascular events like myocardial infarction, angina, and coronary heart disease.

## Materials and methods

Study population

A retrospective cross-sectional study using the National Health and Nutrition Examination Survey (NHANES) database from 2013 to 2018 was performed. The datasets were downloaded from the NHANES website and combined using SAS software (Version 9.4). We included participants aged 12 years and older and identified respondents with a positive history of cardiovascular disease (CVD: myocardial infarction, angina, coronary heart disease, congestive heart failure). Sociodemographic variables such as age, gender, race, and annual household income were included. We excluded participants with missing information on age, race, cardiovascular disease, and smoking. We collected information on self-reported substance use (methamphetamine, cocaine, heroin, marijuana/hashish, injectable steroids, smoking [cigarette, cigar, smokeless tobacco, and e-cigarette]) from our study population. Patient characteristics of interest included: age (12-18, 19-45, 46-65, and 65 and older), gender (male and female), race (Mexican American, other Hispanics, Non-Hispanic White, Non-Hispanic Black, Non-Hispanic Asian, Other Race - Including Multi-Racial), concomitant diagnoses (hypertension, diabetes, hypercholesterolemia, obesity) and annual household income (AHI). Tables 4 and 5 in the appendix describe the definitions for the various concurrent conditions and the relevant questionnaire utilized for this study.

Details about the dataset 

NHANES is a population-based, cross-sectional survey administered by the Centers for Disease Control and Prevention (CDC) to evaluate the health of children and adults in the USA. NHANES datasets are released in two-year cycles and utilize a multistage probability sampling design to create a national representative sample for each cycle. The sampling designs and protocols of NHANES are assessed by the US Department of Health and Human Services and approved by the National Centre for Health Statistics Research Ethics Review Board every year. The NHANES surveys comprise demographic, socioeconomic, dietary, laboratory, and health-related questions. The examination component includes medical, dental, and physiological measurements, as well as laboratory tests administered by highly trained medical personnel. The datasets are available on the CDC website https://www.cdc.gov/nchs/nhanes/about_nhanes.htm. The information on datasets and user guide resources are available on the NHANES website. Since the data is de-identified, informed consent or IRB approval was not required. 

Outcomes

The primary outcome of this study was to evaluate the prevalence of various SUDs amongst respondents diagnosed with CVDs in NHANES datasets between 2013 to 2018. The secondary aim was to find an association between the use of different substances and CVDs.

Statistical analysis

We analyzed the datasets using Statistical Analysis System (SAS) software (Version 9.4). We performed univariate analysis to find demographic characteristics of respondents with CVDs and the association between CVDs and substance use using an unpaired student’s t-test, Mann-Whitney test, and chi-square test. Mix effect multivariable logistic regression models were generated to predict the odds of the presence of different substances amongst respondents with CVDs. The sample size was not pre-decided, and a p-value of <0.05 (alpha criteria) was considered statistically significant. Models were adjusted with demographics (age, gender, race, AHI) and comorbidities (hypertension, diabetes, hypercholesterolemia, obesity). Areas under the receiver operating characteristic curve (ROC) (c-value) for individual models were calculated.

## Results

Population and disease characteristics

From 2013 to 2018, we have identified 263465 respondents with mentioned details on CVDs. Out of these, 20821 (7.90%) respondents had a medical history of CVDs (angina [2.71%] and MI [4.51%]). History of CVDs were more prevalent amongst the population with age group of 66 and above (63.44% vs. 22.68%), male gender (62.23% vs. 45.98%), non-Hispanic White race (54.47% vs. 38.13%), and AHI less than $24,999 (41.80% vs. 25.94%) in comparison to respondents without a history of CVDs (p<0.0001). Respondents with a history of CVDs were found to be older (median age: 69 vs. 50-years old), 66-years and above (19.36% vs. 6.81% [46-65 years] vs. 1.17% [18-45 years]), male (10.40% vs. 5.66% [female]), non-Hispanic White (10.92% vs. 7.69% [other Hispanic] vs. 6.78% [Mexican American]), and with AHI less than $24,999 (12.21% vs. 4.01% [> $100,000]). (p<0.0001) Concurrent history of hypertension (85.98% vs. 79.53%), hypercholesterolemia (68.78% vs. 34.54%), diabetes (37.86% vs. 12.70%), stroke (17.4% vs. 2.71%), and congestive heart failure (28.80% vs. 1.31%) were more prevalent amongst respondents with a history of CVDs in comparison to respondents without a history of CVDs (p<0.0001) (Table 1).

**Table 1:** Demographic characteristics of respondents with cardiovascular disorders (NHANES years 2013-2018) AHI: Annual Household Income; COPD: Chronic obstructive pulmonary disease; LDL: low-density lipoprotein *Calculated by NIH equation 2 (mg/dl); #Column percentage comparison from SAS output

	Cardiovascular Disorder n=20821 (7.90%)#	No-Cardiovascular Disorder n= 242644 (92.10%)#	Total n= 263465 (100%)	p -value
Median Age in Year (Q1-Q3)	69 (61-78)	50 (35-63)		< .0001
Age Groups (%)				< .0001
18-45 years	1173 (5.63)	99469 (40.99)	100642 (38.20)	
46-65 years	6439 (30.93)	88144 (36.33)	94583 (35.90)	
66 and above	13209 (63.44)	55031 (22.68)	68240 (25.90)	
Gender (%)				< .0001
Male	12956 (62.23)	111576 (45.98)	124532 (47.27)	
Female	7865 (37.77)	131068 (54.02)	138933 (52.73)	
Race (%)				< .0001
Mexican American	1877 (9.01)	35473 (14.62)	37350 (14.18)	
Other Hispanic	2140 (10.28)	25697 (10.59)	27837 (10.57)	
Non-Hispanic White	11342 (54.47)	92528 (38.13)	103870 (39.42)	
Non-Hispanic Black	3537 (16.99)	48654 (20.05)	52191 (19.81)	
Asian	1179 (5.66)	31337 (12.91)	32516 (12.34)	
Other Race	746 (3.58)	8955 (3.69)	9701 (3.68)	
AHI (%)				< .0001
$ 0 to $ 24,999	8052 (41.80)	57915 (25.94)	65967 (27.20)	
$25,000 to $64,999	6979 (36.23)	80436 (36.02)	87415 (36.04)	
$65,000 to $99,999	2156 (11.19)	35285 (15.80)	37441 (15.44)	
$100,000 and over	2076 (10.78)	49667 (22.24)	51743 (21.33)	
Comorbidities (%)				
Hypertension	13529 (85.98)	68102 (79.53)	81631 (80.53)	< .0001
Hypercholesterolemia	14321 (68.78)	83806 (34.54)	98127 (37.24)	< .0001
LDL-Cholesterol *Median (Q1-Q3)	91.00 (69-118)	111.00 (89-136)		< .0001
Diabetes	7883 (37.86)	30826 (12.70)	38709 (14.69)	< .0001
Stroke	3627 (17.42)	6574 (2.71)	10201 (3.87)	< .0001
COPD	3488 (16.75)	6804 (2.80)	10292 (3.91)	< .0001
Anemia	1876 (9.01)	9816 (4.05)	11692 (4.44)	< .0001
Cancer	4965 (23.85)	24023 (9.90)	28988(11.00)	< .0001
Liver disease	1986 (9.54)	10938 (4.51)	12924 (4.91)	< .0001
Congestive heart failure	5996 (28.80)	3171 (1.31)	9167 (3.48)	< .0001

Prevalence of substance abuse amongst respondents with CVDs

Using marijuana (CVD vs. no-CVD 65.42% vs. 52.81%; p<0.0001), cigarette smoking (60.47% vs. 40.41%; p<0.0001), cigar-smoking (47.05% vs. 35.58%; p<0.0001), methamphetamine/cocaine/heroin (23.82% vs. 16.71%; p<0.0001), smokeless tobacco use (18.53% vs. 14.59%; p<0.0001), injectable illegal drug use (4.67% vs. 2.43%; p<0.0001) cigarettes without filters (2.28% vs. 1.10%; <0.0001) were prevalent amongst the respondents with a history of CVDs when compared with respondents without a history of CVDs (Table 2).

**Table 2:** Prevalence of substance abuse amongst respondents with Cardiovascular Disorders #Column percentage comparison from SAS output

	Cardiovascular Disorder n= 20821 (7.90%) #	No-Cardiovascular Disorder n= 242644 (92.10%) #	Total n=263465 (100%)	p - value
Methamphetamine/Cocaine/Heroin (%)	2304 (23.82)	31914 (16.71)	34218 (17.05)	< .0001
Alcohol use disorders (%)	3427 (31.01)	76207 (48.07)	79634 (46.96)	< .0001
Marijuana or hashish (%)	2673 (65.42)	79458 (52.81)	82131 (53.14)	< .0001
Injectable illegal drug use (%)	452 (4.67)	4647 (2.43)	5099 (2.54)	< .0001
Cigar smoking (%)	6620 (47.05)	55688 (35.58)	62308 (36.53)	< .0001
Smokeless tobacco (%)	2607 (18.53)	22835 (14.59)	25442 (14.91)	< .0001
E-cigarette (%)	2177 (15.47)	26731 (17.08)	28908 (16.95)	< .0001
Cigarette smoking (%)	12591 (60.47)	98044 (40.41)	110635 (41.99)	< .0001
Cigarette Filter type - No (%)	81 (2.28)	405 (1.10)	486 (1.21)	< .0001
Cigarette Filter type - Yes (%)	3476 (97.72)	36320 (98.90)	39796 (98.79)	< .0001
Number of cigarettes smoked per day	10 (5-20)	8 (4-15)		< .0001

Multivariable regression analysis showing the relationship between CVDs and substance abuse

After adjusting for socio-demographics (age, race, gender, and AHI) and concurrent comorbidities (hypertension, diabetes, hypercholesterolemia, obesity, and stroke), marijuana or hashish (aOR: 1.98; 95% CI: 1.98-1.98), injectable illegal drug use (aOR: 2.15; 95% CI: 2.14-2.15), cigarette smoking (aOR: 1.55; 95% CI: 1.55-1.55), were associated with higher odds of CVDs in comparison without substance abuse (Table 3).

**Table 3:** Multivariable logistic regression analysis showing an association between substance abuse and cardiovascular disorders The model was adjusted for socio-demographics (age, race, gender, and annual household income) and concurrent comorbidities (hypertension, diabetes, hypercholesterolemia, obesity, and stroke).

	Odds ratio (OR)	95% Confidence interval (95%CI)	p-value
Alcohol use disorders (1 vs. 0)	1.17	1.17-1.17	< .0001
Marijuana or hashish (1 vs. 0)	1.98	1.98-1.98	< .0001
Injectable illegal drug (1 vs. 0)	2.15	2.14-2.15	< .0001
Cigarette smoking (1 vs. 3)	1.55	1.55-1.55	< .0001
E-cigarette (1 vs. 0)	2.61	2.61-2.61	< .0001
c - value	0.69		

## Discussion

Multiple risk factors have been identified to be associated with CVDs. Our study has established an association with methamphetamine, cocaine, heroin, injectable illegal drug use, cigar-smoking, cigarette smoking, and cigarette smoking without filters. When compared to patients with non-premature atherosclerotic cardiovascular disease (ASCVD), patients with premature ASCVD had a higher prevalence of all recreational substance use, with female users having a greater risk of premature ASCVD compared with male counterparts. Among all illicit drugs, the use of amphetamines and cannabis were found to have the greatest odds of early-onset ASCVD [17]. In terms of gender, race, household income, and comorbidities, as shown in our study, there were various contributions indicative of a relative correlation amongst substance users.

As per the latest NSDUH compiled by the Substance Abuse and Mental Health Services Administration, among people aged 12 or older in 2019, 57.2 million people were reported to have used illicit drugs in the past year. Interestingly, marijuana was found to have been used by 48.2 million people in the year 2018. Per the same survey, in 2019, among people aged 12 years or older, 60.1% responded to having used a substance during the prior month, where alcohol was found to be more frequently consumed (50.8%), followed by use of tobacco (21.1%), and illicit drugs (13%) [2]. Across all age groups, prevalence rates of alcohol, tobacco, and marijuana use were noted to be higher when compared to other SUDs. While the prevalence rates of marijuana were higher than tobacco in the early adolescent population, it was noted to be reversed in the late adolescent population. [18]. Overall, different age groups experienced different effects of SUDs concerning vascular events.

This retrospective cross-sectional study evaluated the prevalence of different SUDs amongst the population with a history of CVDs and the association of substance use with CVDs. We found that prevalence rates of CVD are higher in the cohort with a history of previous or current SUDs when compared to those who did not have any SUDs. A longitudinal record linkage study done by Thylstrup et al. stated that 828 individuals (4.53%) had a history of CVD at treatment entry out of the 17,642 patients seeking treatment for drug use disorder. And of the remaining 16,820 patients, 1535 were traced and found to be new incident cases of CVD during a mean follow-up time of 7.5 years [19].

With advancing age, the history of CVDs is more prevalent. Markedly the prevalence of CVDs is doubled in the age group 66 and above when compared to 45- 65 years age group respondents (62.3% vs. 37.77%). Prevalence rates for CVD are higher among the non-Hispanic white population. This is contradictory to findings observed in a comprehensive examination of cardiovascular disease in Hispanics in the USA by Rodriguez CJ et al. [20]. In 2010, the American Heart Association declared 2020 health strategy goals to reduce deaths attributable to CVDs and stroke by 20% and to improve the cardiovascular health of all Americans by 20% by focusing on seven key risk factors: smoking, BMI, diet, physical activity, blood pressure, blood glucose, and total cholesterol [21]. Hispanics, especially female Hispanics, are significantly less aware of CVDs as the leading cause of death and their risk factors for CVDs than Non-Hispanic Whites [22]. Hispanic groups are more likely to be uninsured, have low socioeconomic status, and have poor education causing less access to health care and reporting [23]. Therefore, the above-mentioned factors could be the reason to have lesser prevalence rates of CVD in Hispanic Whites compared to non-Hispanic Whites. We also found hypertension to be the most common risk factor associated with CVD. Amongst the responders with CVDs, use of marijuana and cigarette smoking were more prevalent, followed by methamphetamine, cocaine, heroin, and injectable illegal drug use. According to a study done by Azofeifa et al., between 2002 and 2014, there was a 455% increase in marijuana consumption among U.S. adults in the age group of 55-64 and a 333% increase in those older than 64 years, who are the utmost at-risk population for cardiovascular events [24]. A multi-center study done by Mittleman et al. showed that the risk of myocardial infarction onset was elevated 4.8-fold (95% CI, 2.9-9.5; p-value<0.001) within the first hour after smoking marijuana [25].

A retrospective study conducted by Jivanji et al. using the Behavioral Risk Factor Surveillance System (BRFSS) database of the year 2017 showed that odds of having CVDs were significantly high among the users of marijuana when compared to those who did not use marijuana. Furthermore, it was found that higher BMIs, lower income, no exercise, tobacco use, alcohol use, and depression all had a significantly higher prevalence of CVDs among marijuana users [26].

The incidence of cardiovascular disease was twice in the respondents with history of cigarette smoking without filters when compared to the patients with a history of cigarette smoking with filters (16.67% vs. 8.73%; p-value = <0.0001). Smoking is responsible for 14000 deaths annually from premature cardiovascular diseases. It has been established by previous studies that the introduction of filter use might have been associated with less cardiovascular event risk [27]. However, some studies have shown that there were no statistically significant results on the risk of CVDs and/or mortality among the population smoking cigarette with and without filters [28-30]. A cross-sectional survey reported a smoking prevalence of 77% among patients getting treatment for substance use problems [31]. Smoking can contribute to cardiovascular disease by the formation of atheromatous plaques and thrombosis [8,27]. A decline in the rate of recurrent myocardial infarction, recurrent congestive heart failure, and recurrent cardiac arrest after smoking cessation was evident in previous studies [32].

Our study is a large, nationwide population study covering from 2013 to 2018, which included the prevalence of various substance use and its association with cardiovascular disorders in a single study. However, our study has limitations corresponding to recall bias, no data on follow-up, or level of severity of the disease. The study also did not consider abuse details like duration and amount of intake. Hence, more research is required to identify the role of substance use in causing cardiovascular disorders.

## Conclusions

Multiple risk factors have been identified with cardiovascular diseases. Our study has established an association with methamphetamine, cocaine, heroin, injectable illegal drug use, cigar-smoking, e-cigarette smoking, and cigarette smoking without filters. Hence, it is necessary to do further studies to access the risk of CVDs in the population using these substances. According to the American College of Cardiology and American Heart Association (ACC-AHA) guidelines, cigarette smoking is a vital independent risk factor for atherosclerotic cardiovascular disease and premature death. There is a need for further research to address the use of filters as a risk factor for CVDs in the high-risk population (i.e., in patients with a history of hypertension and diabetes).

Many complications of substance use disorders are preventable and reversible; it is very crucial to screen for these in patients with cardiovascular disease. All physicians must recognize the rise in cardiovascular events over the past decade and remain alert in counseling their patients accordingly. It is important to bring awareness among the general population as it is the first step toward the prevention of any disease. Strict guideline on the legalization of substance usage and accessibility is necessary.

## Appendices

Table 4

**Table 4:** NHANES variables/Questionnaire utilized for our study and their Descriptions

Demographic variables/ Questionnaire	Description
RIDAGEYR	Age in years at screening
RIAGENDR	Gender
RIDRETH1	Race/Hispanic origin
INDHHIN2	Annual household income
WHQ030	How do you consider your weight
DIQ010	Doctor told you have diabetes
BPQ080	Doctor told you - high cholesterol level
BPQ020	Ever told you had high blood pressure
ALQ111	Ever had a drink of any kind of alcohol
DUQ200	Ever used marijuana or hashish
DUQ250	Ever use any form of cocaine
DUQ290	Ever used heroin
DUQ330	Ever used methamphetamine
SMQ910	Ever used smokeless tobacco?
SMQ621	Cigarettes smoked in entire life
SMQ900	Ever used an e-cigarette?
DUQ370	Ever use a needle to inject illegal drug
DUQ380A	Drugs injected - Cocaine
DUQ380B	Drugs injected - Heroin
DUQ380C	Drugs injected - Methamphetamine
DUD380F	Drugs injected - Steroids or other drugs
MCQ160b	Ever told had congestive heart failure
MCQ160c	Ever told you had coronary heart disease
MCQ160d	Ever told you had angina/angina pectoris
MCQ160e	Ever told you had a heart attack
MCQ160f	Ever told you had a stroke

Table 5

**Table 5:** NHANES Questionnaire utilized for our study in regards to Cardiovascular Disorders (CVD) and Substance Use disorder (SUD) SP: Sample Participant

CVD/SUD	Relevant Question(s)
CVD Q1	Has a doctor or other health professional ever told {you/SP} that {you/s/he} . . .had coronary (kor-o-nare-ee) heart disease?
CVD Q2	Has a doctor or other health professional ever told {you/SP} that {you/s/he} . . .had a heart attack (also called myocardial infarction (my-o-car-dee-al in-fark-shun))?
CVD Q3	Has a doctor or other health professional ever told {you/SP} that {you/s/he} . . .had angina (an-gi-na), also called angina pectoris?
Alcohol use disorder	Heavy drinking is defined as consuming 8 or more drinks per week in females and 15 or more drinks per week in males
Smoking Q1	Smoked at least 100 cigarettes in life
Smoking Q2	Have {you/SP} ever used an e-cigarette even once?
Smoking Q3	{Have you/Has SP} ever used smokeless tobacco even once? (Smokeless tobacco products are placed in the mouth and nose and include chewing tobacco, snuff, dip, snus (pronounced as "snoose"), and dissolvable tobacco.)
Smoking Q4	{Have you/Has SP} ever smoked a regular cigar, cigarillo, or little filtered cigar even one time?
Smoking Q5	During the past 30 days, on the days that {you/SP} smoked, about how many cigarettes did {you/s/he} smoke per day?
Smoking Q6	CIGARETTE PRODUCTS FILTERED OR NON-FILTERED
Methamphetamine	Have you ever, even once, used methamphetamine?
Cocaine	Have you ever, even once, used cocaine, in any form?
Heroin	Have you ever, even once, used heroin?
Marijuana or hashish	Have you ever, even once, used marijuana or hashish?
Injectable illegal drug	Which of the following drugs (cocaine, heroin, methamphetamine, steroids) have you injected using a needle?

## References

[1] Virani SS, Alonso A, Benjamin EJ (2020). Heart disease and stroke statistics-2020 update: A report from the American Heart Association. Circulation.

[2] SAMSHA SAMSHA (2019). Key substance use and mental health indicators in the United States: Results from the 2019 National Survey on Drug Use and Health. HHS Publ No PEP19-5068. NSDUH Ser H-54.

[3] Qureshi AI, Suri MF, Guterman LR, Hopkins LN (2001). Cocaine use and the likelihood of nonfatal myocardial infarction and stroke: data from the Third National Health and Nutrition Examination Survey. Circulation.

[4] Mukamal KJ, Maclure M, Muller JE, Mittleman MA (2008). An exploratory prospective study of marijuana use and mortality following acute myocardial infarction. Am Heart J.

[5] DeFilippis EM, Singh A, Divakaran S (2018). Cocaine and marijuana use among young adults with myocardial infarction. J Am Coll Cardiol.

[6] Roth GA, Mensah GA, Johnson CO (2020). Global burden of cardiovascular diseases and risk factors, 1990-2019: Update from the GBD 2019 study. J Am Coll Cardiol.

[7] Piano MR (2017). Alcohol’s effects on the cardiovascular system. Alcohol Res.

[8] Larsson SC, Orsini N, Wolk A (2015). Alcohol consumption and risk of heart failure: a dose-response meta-analysis of prospective studies. Eur J Heart Fail.

[9] Torrisi M, Pennisi G, Russo I (2020). Sudden cardiac death in anabolic-androgenic steroid users: A literature review. Medicina (Kaunas).

[10] Baggish AL, Weiner RB, Kanayama G, Hudson JI, Lu MT, Hoffmann U, Pope HG Jr (2017). Cardiovascular toxicity of illicit anabolic-androgenic steroid use. Circulation.

[11] Lange RA, Hillis LD (2001). Cardiovascular complications of cocaine use. N Engl J Med.

[12] Kevil CG, Goeders NE, Woolard MD (2019). Methamphetamine use and cardiovascular disease. Arterioscler Thromb Vasc Biol.

[13] Aryana A, Williams MA (2007). Marijuana as a trigger of cardiovascular events: speculation or scientific certainty?. Int J Cardiol.

[14] Csordas A, Bernhard D (2013). The biology behind the atherothrombotic effects of cigarette smoke. Nat Rev Cardiol.

[15] Theron AJ, Feldman C, Richards GA, Tintinger GR, Anderson R (2019). Electronic cigarettes: where to from here?. J Thorac Dis.

[16] Benowitz NL, Burbank AD (2016). Cardiovascular toxicity of nicotine: Implications for electronic cigarette use. Trends Cardiovasc Med.

[17] Mahtta D, Ramsey D, Krittanawong C (2021). Recreational substance use among patients with premature atherosclerotic cardiovascular disease. Heart.

[18] Schulte MT, Hser YI (2014). Substance use and associated health conditions throughout the lifespan. Public Health Rev.

[19] Thylstrup B, Clausen T, Hesse M (2015). Cardiovascular disease among people with drug use disorders. Int J Public Health.

[20] Rodriguez CJ, Allison M, Daviglus ML (2014). Status of cardiovascular disease and stroke in Hispanics/Latinos in the United States: a science advisory from the American Heart Association. Circulation.

[21] Lloyd-Jones DM, Hong Y, Labarthe D (2010). Defining and setting national goals for cardiovascular health promotion and disease reduction: the American Heart Association's strategic Impact Goal through 2020 and beyond. Circulation.

[22] Mosca L, Ferris A, Fabunmi R, Robertson RM (2004). Tracking women's awareness of heart disease: an American Heart Association national study. Circulation.

[23] Baum S, Flores SM (2011). Higher education and children in immigrant families. Future Child.

[24] Azofeifa A, Mattson ME, Schauer G, McAfee T, Grant A, Lyerla R (2016). National estimates of marijuana use and related indicators - National Survey on Drug Use and Health, United States, 2002-2014. MMWR Surveill Summ.

[25] Mittleman MA, Lewis RA, Maclure M, Sherwood JB, Muller JE (2001). Triggering myocardial infarction by marijuana. Circulation.

[26] Zhang C, Qin YY, Chen Q (2014). Alcohol intake and risk of stroke: a dose-response meta-analysis of prospective studies. Int J Cardiol.

[27] Castelli WP, Dawber TR, Feinleib M, Garrison RJ, Mcnamara PM, Kannel WB (1981318). The Filter Cigarettes and Coronary Heart Disease: The Framingham Study.

[28] Hawthorne VM, Fry JS (1978). Smoking and health: the association between smoking behaviour, total mortality, and cardiorespiratory disease in west central Scotland. J Epidemiol Community Health (1978).

[29] Lee PN, Garfinkel L (1981). Mortality and type of cigarette smoked. J Epidemiol Community Health.

[30] Smith PH, Mazure CM, McKee SA (2014). Smoking and mental illness in the US population. Tob Control.

[31] Kelly PJ, Baker AL, Deane FP, Kay-Lambkin FJ, Bonevski B, Tregarthen J (2012). Prevalence of smoking and other health risk factors in people attending residential substance abuse treatment. Drug Alcohol Rev.

[32] Jivanji D, Mangosing M, Mahoney SP, Castro G, Zevallos J, Lozano J (2020). Association between marijuana use and cardiovascular disease in US adults. Cureus.

